# Association Between Migraine With and Without Aura and Cervical Artery Dissection

**DOI:** 10.7759/cureus.87883

**Published:** 2025-07-14

**Authors:** Robert J Trager, Zachary A Cupler, Timothy J Williamson, Eric Chun-Pu Chu

**Affiliations:** 1 Chiropractic Medicine, Connor Whole Health, University Hospitals Cleveland Medical Center, Cleveland, USA; 2 Family Medicine and Community Health, Case Western Reserve University School of Medicine, Cleveland, USA; 3 Biostatistics and Bioinformatics, Clinical Research Training Program, Duke University School of Medicine, Durham, USA; 4 Physical Medicine and Rehabilitative Services, Butler Veterans Affairs (VA) Health Care System, Butler, USA; 5 Institute for Clinical Research Education, University of Pittsburgh, Pittsburgh, USA; 6 UCHealth Spine Center, Anschutz Medical Campus (AMC), University of Colorado Hospital, Aurora, USA; 7 New York Medical Group, EC Healthcare, Hong Kong, CHN

**Keywords:** carotid artery dissection, cerebrovascular disorders, headache, migraine, real-world data, vertebral artery dissection

## Abstract

Introduction: Research suggests that migraine without aura (MwoA) is a risk factor for cervical artery dissection (CeAD); however, the association between migraine with aura (MwA) and CeAD remains inconclusive. We tested the coprimary hypotheses of positive associations between both MwA and MwoA and CeAD within two years following migraine diagnosis, compared to matched non-migraine controls, as measured by risk ratio (RR).

Methods: We queried de-identified US medical records data (TriNetX, LLC, Cambridge, Massachusetts, United States) spanning the years 2014-2024 to identify three cohorts of patients without previous CeAD. These included (1) individuals with a new diagnosis of MwA, (2) individuals with a new diagnosis of MwoA, and (3) non-migraine controls. Controlling for confounders, we created two 1:1 propensity-matched cohort pairs, having 217,390 patients (MwA versus controls) and 277,865 patients (MwoA versus controls).

Results: Compared to non-migraine controls, patients with either MwA or MwoA had an increased incidence and risk of CeAD: MwA (95% CI) (0.095% vs. 0.016%; RR=5.91 (4.13,8.46); p<0.0001) and MwoA (0.058% vs. 0.016%; RR=3.60 (2.59,5.01); p<0.0001).

Conclusion: Incidence and risk of vertebral and carotid artery dissections were similarly increased for both MwA and MwoA. Further research is needed to examine the pathophysiological mechanisms underlying the migraine-CeAD association, potential mediating factors, and temporal association between migraine attacks and CeAD.

## Introduction

Cervical artery dissection (CeAD) is a serious condition characterized by a tear in the arterial walls in the neck and can lead to stroke. Its incidence is 8.9 per 100,000 person-years [1], and while it can occur at any age, it has a mean age of 47 [2]. Migraine, a common neurological disorder peaking at 13% prevalence in middle age, often begins in adolescence and is more common in women [3]. Migraine can be classified into two types, namely, migraine without aura (MwoA) and migraine with aura (MwA), with aura typically presenting as visual disturbances [4]. Approximately 28% of migraineurs experience aura [4]. Migraine in general is a risk factor for myocardial infarction and stroke, while MwA is a risk factor for cardiovascular mortality [5].

Several studies have examined the association between MwA and CeAD, yielding conflicting results. A recent systematic review including 9,857 patients found a positive association between MwoA and CeAD but no significant association between MwA and CeAD, a finding which was limited by substantial heterogeneity [6]. An earlier systematic review in 2011 yielded similar results [7]. Conversely, a recent registry study including 1,468 CeAD patients found a positive association between CeAD and both MwA and MwoA [2]. Given the discrepancy regarding a possible association between MwA and CeAD, there is a need to clarify this relationship.

The existing literature has several limitations. Several case-control studies have been prone to recall bias, inadequate documentation of migraine history, or reliance upon a self-reported migraine status [6,7]. Moreover, the variable nature of migraine auras can lead to their underdiagnosis [8], thereby potentially leading to the misclassification of migraine subtype. Consistent with the prevalence of migraine subtypes, studies have included fewer patients with MwA compared to MwoA, potentially affecting the statistical power of previous systematic reviews [7].

This study tested the coprimary hypotheses that MwA and MwoA are associated with an increased risk of CeAD. We used a retrospective cohort design to compare the risk of CeAD in adult and pediatric patients with diagnosed MwA or MwoA over two years after migraine diagnosis to matched non-migraine controls (i.e., MwA versus controls and MwoA versus controls).

## Materials and methods

Study design

This study used a retrospective cohort design to examine the relative risk of CeAD among patients with MwA or MwoA and adhered to a protocol registered in Open Science Framework (https://osf.io/9g8wt) [9]. The data range was updated to the previous 10 years preceding the query date of August 2, 2024, to minimize potential biases related to increasing incidence and detection of CeAD [2]. Data were sourced from TriNetX (TriNetX, LLC, Cambridge, Massachusetts, United States), a network encompassing over 129 million patients across 91 US medical centers and their affiliated healthcare offices [10]. The TriNetX platform adheres to data privacy regulations, including those of the Health Insurance Portability and Accountability Act, and is International Organization for Standardization 27001:2013-certified. The University Hospitals Institutional Review Board (IRB) (Cleveland, Ohio, United States) considers studies using de-identified data from the online TriNetX platform to represent "Not Human Subjects Research" and exempted the present study from IRB review, waiving the need for consent (STUDY20250827).

Data available within TriNetX include standardized medical codes (e.g., International Classification of Diseases (ICD-10) and Current Procedural Terminology (CPT)), with the software automatically converting ICD-10 to ICD-9 when needed. TriNetX monitors the consistency, conformance, and completeness of data quality, demonstrating at least 87% medication data completeness [11]. This study used natural language processing software embedded within the TriNetX platform (Averbis, Freiburg im Breisgau, Germany [10]). This software extracts data from unstructured text within clinical charts and test results and has reliability and accuracy comparable to manual chart review [12-14]. Diagnoses, including MwA, MwoA, and CeAD, included in the analysis represent those appended by clinicians within healthcare organizations participating with TriNetX as part of real-world clinical practice.

Participants

We included individuals at least age 10, with no upper age limit, and divided them into cohorts: (1) those with a new diagnosis of MwA (ICD-10: G43.1), (2) those with a new diagnosis of MwoA (ICD-10: G43.0), and (3) non-migraine controls undergoing the first instance of an adult or pediatric general examination (ICD-10: Z00.0 and Z00.1, respectively) coded in the primary priority position. A focus on new migraine diagnoses aimed to standardize the duration of migraine symptoms and related care and to avoid including long-term migraine sufferers who may have already experienced CeAD. As a measure of ensuring adequate follow-up, we required one additional healthcare visit over the two-year follow-up window in all cohorts. To improve data completeness, we required all individuals to have body mass index and systolic and diastolic blood pressure measurements within three years on or before the index date. We excluded individuals with previous CeAD or cervical arterial injury and related surgeries. For patients in the non-migraine control cohort, we excluded individuals with any type of migraine (ICD-10: G43) or migraine-specific medications (Appendix 1).

Variables

We used propensity score matching to balance confounding variables between cohorts which have a known association with CeAD to minimize bias [15], considering variables within three years preceding and including the index date including sex, body mass index, blood pressure, pregnancy, and various comorbidities [16,17] (Appendix 2). We also propensity matched on upper respiratory infections and quinolones, which have been associated with an increase in CeAD in prior studies [2,18]. We used an aggregated parent ICD-10 code to define and match on adverse socioeconomic and psychosocial circumstances based on previous work [19]. To minimize potential temporal bias from different years of cohort entry, we matched patients by their age at the index date (migraine diagnosis or examination) and their current age (at the time of our query). This approach aimed to minimize confounding related to the increasing detection of CeAD in more recent years [1], helping ensure similar age distributions and years of exposure across compared cohorts.

Primary outcomes

We ascertained diagnoses of CeAD using ICD-10 codes (I77.71: carotid artery dissection and I77.74: vertebral artery dissection) bolstered by natural language processing, over a two-year follow-up window beginning the day after the index date of inclusion. A shorter follow-up window was avoided as it may yield insufficient events, while a longer follow-up would diminish available sample size and/or increase bias by requiring a further retrospective data range. We avoided examining stroke as an outcome, as not all instances of CeAD result in stroke [20]. Additionally, stroke has different risk factors compared to CeAD [2] and would require different selection criteria and matched covariates [15].

Secondary outcomes

We explored the cumulative incidence of CeAD per cohort and calculated separate RRs for carotid and vertebral artery dissection after matching. We also described the proportion of migraine cohorts who were prescribed antimigraine medications during follow-up (Anatomical Therapeutic Chemical: N02C (antimigraine preparations) or Veterans Affairs: CN105 (antimigraine agents), including ergot alkaloids, triptans, calcitonin gene-related peptide antagonists, gepants, and others).

Statistical methods

We used the TriNetX platform software to compare baseline characteristics, using independent samples t-tests and Pearson's chi-squared tests. TriNetX applies logistic regression to pooled covariate matrices to calculate propensity scores using Python (scikit-learn version 1.3, Python Software Foundation, Wilmington, Delaware, United States). Propensity scores of 1 indicate the greatest likelihood of being in the non-migraine control cohorts. Patients were matched 1:1 using greedy nearest-neighbor matching with a caliper of 0.1 of pooled standard deviations, forming two separate pairs of cohorts (MwA versus controls and MwoA versus controls). We evaluated covariate balance using standardized mean difference (SMD) with values greater than 0.1 representing residual imbalance [21]. We reported the mean data points (i.e., facts, diagnoses, test results, etc.) per patient as a marker of data density. The RRs for CeAD were calculated after matching by dividing the incidence in the MwA or MwoA cohorts by the incidence in their respective matched control cohort. We used a Bonferroni-adjusted p-value of 0.025 to account for coprimary RR outcomes. We plotted propensity score densities and cumulative incidence of CeAD with 95% confidence intervals using R (version 4.2.2, R Foundation for Statistical Computing, Vienna, Austria) and ggplot2.

We calculated a total required sample size for both analytic models (i.e., MwA versus controls and MwoA versus controls) of 234,848 using G*Power (Heinrich-Heine-Universität Düsseldorf, Düsseldorf, Germany) z-tests. Based on data from previous studies [1,6], we used a proportion between cohorts of 0.0002 versus 0.0005 for our primary outcomes using a power of 0.95, an allocation ratio of one, and a two-tailed α error of 0.025. Considering the incidence of CeAD being approximately nine per 100,000 person-years, over a two-year follow-up window, this would equate to 18 events in a cohort of 100,000 individuals or an incidence proportion of 0.00018 (we rounded this value up to 0.0002) [1]. A prior study identified a positive association between migraine and CeAD with an odds ratio of 1.74 [6]. Accordingly, our sample size estimate was conservative and powered to detect an even larger difference in CeAD incidence proportions among migraineurs, of more than double the control cohort.

## Results

Patients

Before propensity matching, there were 240,583 patients in the MwA cohort, 308,972 in the MwoA cohort, and 1,029,852 in the non-migraine control cohort. After matching, the number of patients in each cohort reduced (MwA versus controls: 217,390 patients each; MwoA versus controls: 277,865 patients each). The resulting post-matching non-migraine control cohorts varied slightly in size across each comparison due to the matching strategy, which trimmed non-matching patients when matching to the MwA or MwoA cohort, respectively. After matching, all covariates were optimally matched (SMD<0.1; Table 1).

**Table 1:** Cohort characteristics after matching NA: not applicable; MwA: migraine with aura; MwoA: migraine without aura; SMD: standardized mean difference

	MwA versus controls			MwoA versus controls		
Variable (n (%) or mean (SD))	MwA	Controls	SMD	MwoA	Controls	SMD
N	217,390	217,390	NA	277,865	277,865	NA
Age at index	40.9 (17.4)	42.1 (18.5)	0.066	38.9 (16.8)	39.6 (18.1)	0.040
Age at query (current age)	46.6 (17.5)	47.7 (18.5)	0.063	44.7 (17.0)	45.4 (18.1)	0.037
Female	174581 (80%)	174637 (80%)	0.001	225204 (81%)	225874 (81%)	0.006
Male	42704 (20%)	42651 (20%)	0.001	52554 (19%)	51889 (19%)	0.006
Acute upper respiratory infections	56175 (26%)	54355 (25%)	0.019	72075 (26%)	70070 (25%)	0.017
Adverse socioeconomic and psychosocial circumstances	15075 (7%)	14002 (6%)	0.020	15576 (6%)	15625 (6%)	0.008
Alpha-1-antitrypsin deficiency	91 (<1%)	81 (<1%)	0.002	106 (<1%)	80 (<1%)	0.005
Aortic aneurysm and dissection	1820 (1%)	1684 (1%)	0.007	1748 (1%)	1595 (1%)	0.007
Arterial fibromuscular dysplasia	113 (<1%)	111 (<1%)	<0.001	113 (<1%)	107 (<1%)	0.001
Autoimmune thyroiditis	2680 (1%)	2896 (1%)	0.009	2967 (1%)	3189 (1%)	0.008
Blood pressure, diastolic (mm Hg)	74.3 (11.0)	73.9 (10.4)	0.036	74.5 (10.9)	73.6 (10.4)	0.082
Blood pressure, systolic (mm Hg)	121.5 (16.4)	121.3 (16.0)	0.015	121.3 (16.0)	120.4 (15.8)	0.055
Body mass index (kg/m^2^)	28.9 (7.6)	28.9 (7.7)	0.003	29.1 (7.9)	28.7 (7.8)	0.055
Diabetes mellitus	20971 (10%)	20051 (9%)	0.014	25664 (9%)	24530 (9%)	0.014
Diseases of arteries, arterioles, and capillaries	16485 (8%)	17022 (8%)	0.009	17024 (6%)	16937 (6%)	0.001
Ehlers-Danlos syndromes	495 (<1%)	445 (<1%)	0.005	415 (<1%)	474 (<1%)	0.005
Elevated C-reactive protein	794 (<1%)	811 (<1%)	0.001	878 (<1%)	874 (<1%)	<0.001
Elevated erythrocyte sedimentation rate	650 (<1%)	685 (<1%)	0.003	792 (<1%)	799 (<1%)	<0.001
Elevated white blood cell count	5833 (3%)	5991 (3%)	0.004	6610 (2%)	6669 (2%)	0.001
Family history of cardiovascular disease	18834 (9%)	18974 (9%)	0.002	18620 (7%)	18353 (7%)	0.004
Hyperhomocysteinemia	161 (<1%)	164 (<1%)	0.001	164 (<1%)	153 (<1%)	0.002
Hyperlipidemia	34989 (16%)	35923 (17%)	0.012	38623 (14%)	37592 (14%)	0.011
Hypertensive diseases	53844 (25%)	53629 (25%)	0.002	62389 (22%)	61111 (22%)	0.011
Osteogenesis imperfecta	39 (<1%)	29 (<1%)	0.004	50 (<1%)	26 (<1%)	0.007
Other congenital syndromes affecting multiple systems	484 (<1%)	440 (<1%)	0.004	572 (<1%)	583 (<1%)	0.001
Pregnancy, childbirth, and the puerperium	17659 (8%)	18591 (9%)	0.016	19155 (7%)	19933 (7%)	0.011
Quinolones	25686 (12%)	26895 (12%)	0.017	32158 (12%)	33261 (12%)	0.012
Substance use disorders	34367 (16%)	35386 (16%)	0.013	40366 (15%)	41397 (15%)	0.010
Tobacco use	16439 (8%)	16645 (8%)	0.004	17024 (6%)	16937 (6%)	0.001

Data quality

Before matching, the mean number of data points per patient per cohort was adequate (MwA: 3,017; MwoA: 2,986; non-migraine controls: 1,626). An adequate proportion of each cohort had a length of record spanning at least two years (MwA: 94%; MwoA: 95%; non-migraine controls: 93%). After matching, the proportion of patients with "unknown age" was approximately 0% in all cohorts. Following matching, propensity score densities closely overlapped among paired cohort comparisons (MwA versus non-migraine controls and MwoA versus non-migraine controls), suggesting adequate covariate balance (Appendix 3 and Appendix 4). These findings suggested there were negligible between-cohort differences in covariate balance, data density, and completeness.

Primary outcomes

After matching, the incidence and risk of CeAD were greater among those with MwA compared to non-migraine controls (95% CI) (0.095% vs. 0.016%; RR=5.91 (4.13,8.46); p<0.0001). Similarly, the incidence and risk of CeAD were greater among those with MwoA compared to non-migraine controls (0.058% vs. 0.016%; RR=3.60 (2.59,5.01); p<0.0001). Primary outcomes are summarized in Table 2.

**Table 2:** Incidence and risk ratio of CeAD after matching CeAD: cervical artery dissection; CI: confidence intervals; MwA: migraine with aura; MwoA: migraine without aura; *: primary outcome

	MwA versus controls		MwoA versus controls	
Outcome	MwA	Control	MwoA	Control
Number of patients	217,390	217,390	277,865	277,865
CeAD n (%)	207 (0.095%)	35 (0.016%)	162 (0.058%)	45 (0.016%)
CeAD n per 100,000 person-years	47.6	8.1	29.2	8.1
CeAD risk ratio (95% CI)*	5.91 (4.13,8.46); p<0.0001	Reference	3.60 (2.59,5.01); p<0.0001	Reference

Secondary outcomes

**Figure 1:**
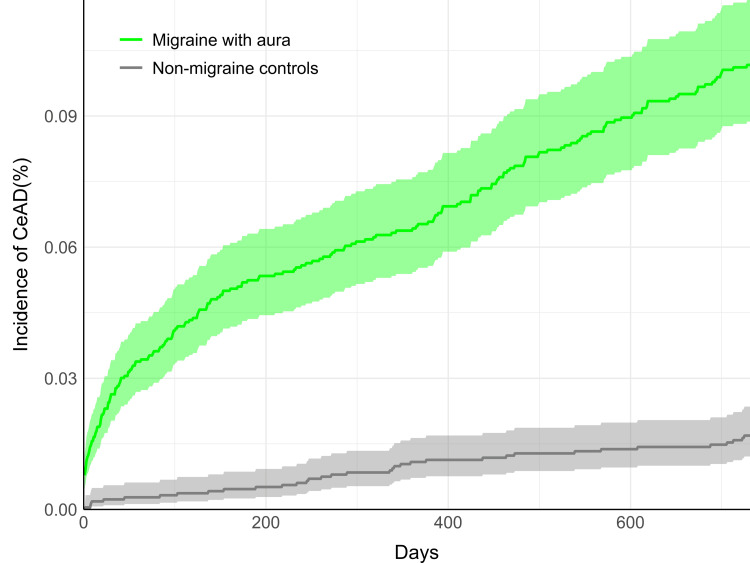
Cumulative incidence of CeAD among patients with migraine with aura (green) compared to non-migraine controls (gray) Results are shown over the two-year follow-up period. Shaded regions indicate 95% confidence intervals. CeAD: cervical artery dissection

**Figure 2:**
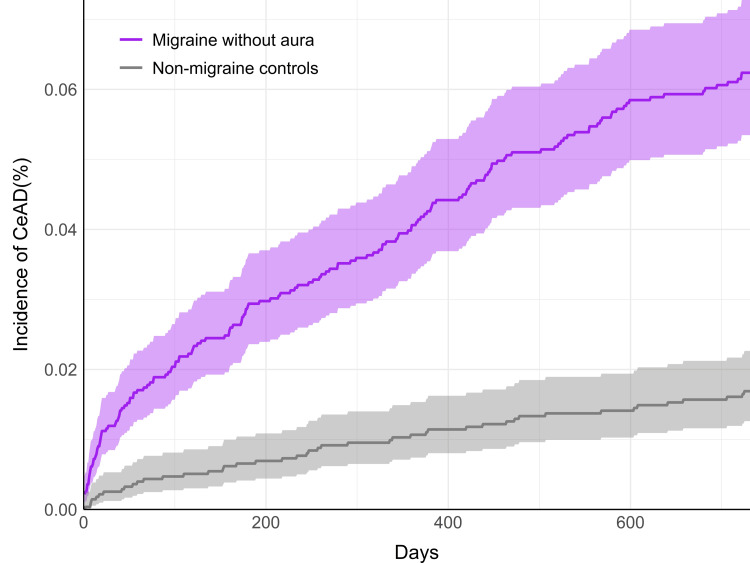
Cumulative incidence of CeAD among patients with migraine without aura (purple) compared to non-migraine controls (gray) Results are shown over the two-year follow-up period. Shaded regions indicate 95% confidence intervals. CeAD: cervical artery dissection

Cumulative incidence plots demonstrated an early increase in CeAD incidence among those with MwA (Figure 1) and MwoA (Figure 2) soon after the index date, demonstrating a curvilinear pattern. In contrast, the CeAD incidence adhered to a relatively linear pattern in both control cohorts.

Outcome-negative control point RR estimates further suggested that cohorts were well balanced after matching. When comparing those with MwA to non-migraine controls, this included azithromycin prescription (95% CI) (11.4% vs. 11%; RR=1.04 (1.02,1.05)) and urinalysis (26.6% vs. 25.5%; RR=1.04 (1.03,1.05)). Comparing the MwoA cohort to non-migraine controls revealed similar findings for azithromycin prescription (11.61% vs. 10.8%; RR=1.07 (1.06,1.09)) and urinalysis (24.9% vs. 24.4%; RR=1.02 (1.01,1.03)).

After matching, comparing those with MwA to non-migraine controls, the incidence and risk of individual subtypes of CeAD were greater, including vertebral artery dissection (95% CI) (123 vs. 20 patients; 0.057% vs. 0.009%; RR=6.15 (3.83,9.87); p<0.0001) and carotid artery dissection (91 vs. 17 patients; 0.042% vs. 0.008%; RR=5.35 (3.19,8.98); p<0.0001).

Similarly, after matching, comparing those with MwoA to non-migraine controls, the incidence and risk of individual subtypes of CeAD were greater, including vertebral artery dissection (91 vs. 27 patients; 0.033% vs. 0.010%; RR=3.37 (2.19,5.18); p<0.0001) and carotid artery dissection (77 vs. 20 patients; 0.028% vs. 0.007%; RR=3.85 (2.35,6.30); p<0.0001).

The proportion and mean prescription count of migraine medications during follow-up were substantial in both migraine cohorts. In the MwA cohort, 32% of patients were prescribed migraine medications (standard deviation) (mean count=5.2 (13.5); median=2). In the MwoA cohort, 38% of patients were prescribed migraine medications (standard deviation) (mean count=5.6 (14.4); median=2).

## Discussion

The present findings support our hypotheses that both MwA and MwoA are associated with an increased risk of CeAD compared to propensity-matched non-migraine controls, including over 400,000 patients and over 800,000 person-years of observation per each analysis in total. Furthermore, MwA and MwoA were associated with an increase in risk of both carotid and vertebral artery dissection. Our identified effect estimates, which were statistically significant and relatively large in magnitude, underscore the clinical relevance of these findings, especially considering that 29-65% of CeADs result in stroke [20,22].

Although a longer follow-up window may yield better long-term insights regarding migraine-CeAD associations, it was avoided in the present study due to methodological concerns. Specifically, biases could arise related to loss to follow-up or other potential intervening factors (e.g., motor vehicle collisions) that could cause CeAD independently and were not possible to account for in the matching strategy. Additionally, extending the follow-up would decrease the available sample size due to a shrinking eligibility window and reduce statistical precision. Our choice of a two-year window was informed by a sample size calculation based on the natural history of CeAD and a pooled risk estimate from a previous study [1,6]. However, future studies should attempt to capture even longer patient data windows, for example, by using patient registries or case-control designs that account for total migraine duration in years.

There were approximately 44% MwA patients when considering all patients in both migraine cohorts. This value is at the upper end of the expected range, as, in general, a mean 28% of migraineurs experience auras (range: 11.9-44.3% across 10 studies) [4]. However, we discourage readers from over-interpreting the proportion of MwA patients in the present study. First, both migraine cohorts were highly selected from large healthcare organizations, having requirements for previous blood pressure and body mass index measurements, among other factors, to maximize data completeness, which potentially skewed their relative representation. Additionally, in real-world datasets, clinicians may use unspecified headache diagnosis codes rather than more specific MwA or MwoA codes (e.g., ICD-10: R51) [23], and patients with unclassified headaches were not included in the present study. Overall, our study was not geared towards representing a specific proportion of either MwA or MwoA, yet was optimized to maximize sample size and data quality in both migraine cohorts for the purposes of our CeAD hypothesis testing.

This study combined analyses for pediatric and adult patients since migraines often begin in adolescence [3], and we aimed to allow our query to capture individuals seeking care during this stage of life. Additionally, combining age groups maximized sample size, which was essential given the low incidence of CeAD. We propensity matched for age in both cohort comparisons, thereby accounting for it statistically. Despite our eligibility criteria allowing inclusion of individuals aged 10 and older, the mean ages in our migraine cohorts (MwA and MwoA) indicate that our findings predominantly reflect the adult population. Future studies could explore age subgroups separately to determine if CeAD risk varies by age among migraineurs. Such investigations could employ regression models to examine the interaction between age, migraine subtypes, and CeAD risk while controlling for other covariates.

The mean ages in our migraine cohorts were 40.9 (SD=17.4) for MwA and 38.9 (SD=16.8) for MwoA, which is at the upper end of the range of expected migraine onset and/or diagnosis. In the United States, migraines increase in prevalence between adolescence and the fourth decade of life [3]. Reasons for our observed slightly higher age range among migraineurs include both the extensive eligibility criteria and reliance upon a real-world dataset. While patients may have had migraines prior to diagnosis, we did not include self-reported migraines or health records not connected to TriNetX, instead relying on clinician-recorded diagnoses for inclusion. Despite allowing for the inclusion of adolescents, our findings better represent adults and may not generalize to adolescents or those at the onset of first-ever migraine.

A direct comparison of our findings with prior studies is precluded by our selection criteria, recent data range, and propensity matching strategy. However, both the MwA and MwoA cohorts had incidences per 100,000 person-years that exceeded a previously reported value for the non-migraine general population (i.e., 8.9 per 100,000 person-years [1]), while the control incidences were comparable to this value (i.e., 8.1). The female predominance of both our MwA and MwoA cohorts aligns with previous data on the topic [3]. Finally, the percentage of migraineurs in the current study prescribed migraine-specific medications (MwA: 32%; MwoA: 38%) is similar to data from a US study which reported that in 2020, the percentage of migraine patients taking either triptans, ergotamine, or cyclic calcitonin gene-related peptide medications was 42.7% [24].

While the association between MwA and CeAD was unclear in previous research [6], our observed significant, positive association may be explained by methodological differences. By using a national dataset, we obtained a large sample size for both migraine subtypes, overcoming a common issue in previous studies of including more patients with MwoA than MwA [7]. A 2023 systematic review examining the migraine-CeAD association included 9,857 total patients across 11 studies [6]. In comparison, each pair of cohorts in the present study included over 400,000 patients, potentially resulting in narrower confidence intervals and more precise effect estimates. Earlier studies often used a case-control design with stroke controls, potentially confounding the association with CeAD due to the known relationship between migraine and stroke [6]. In contrast, our use of non-stroke, non-migraine control cohorts may better examine the migraine-CeAD association. Aside from these differences, our study corroborates previous meta-analyses which identified a positive association between MwoA and CeAD [6,7].

The mechanisms that underlie the migraine-CeAD association remain unclear, although several warrant consideration. Migraine pathophysiology involves interrelated vascular dysfunction, inflammation, central sensitization, and activation of the trigeminal nerve system [25,26]. Neurons activated during a migraine release vasoactive peptides, which could contribute to vascular dysfunction [26]. For example, a recent cohort study involving 210 migraineurs identified significant structural changes in cervical arteries during active migraines via sonography, specifically, an increase in the diameter of the internal carotid artery and a decrease in the diameter of the vertebral artery [27]. Migraineurs, regardless of subtype, are more likely to have structural craniocervical arterial differences, such as decreased blood flow and hypoplasia of the vertebral artery [28], and increased intima-media thickness of the carotid artery compared to non-migraineurs [29]. Additionally, genetic factors may play a role, with specific genes related to vascular function being implicated in both migraine and CeAD [30]. These underlying structural and genetic differences from the non-migraine population may contribute to the association between migraine and CeAD and highlight the need for further investigation into this topic.

The association between both MwA and MwoA and CeAD identified in this study may assist clinicians in recognizing patients at increased risk for CeAD. For clinicians who frequently encounter patients with migraines, such as neurologists, primary care physicians, and chiropractors [3,31], this association may influence patient management strategies. This could include a lower threshold to refer for emergency care and/or consideration of vascular imaging techniques, such as cervical artery sonography or computed/magnetic resonance angiography, to evaluate for CeAD [32]. As supported by our cumulative incidence plots, CeAD diagnosis often occurred soon after a new migraine diagnosis, highlighting the potential relevance of clinical vigilance for the evolving nature of CeAD among such patients. By itself, a new diagnosis of migraine is not enough to suspect CeAD; rather, a prudent patient history and physical examination indicating a pattern suspicious for CeAD is critical to patient management and resource management.

In addition to recognizing the baseline risk associated with migraines, clinicians should also be vigilant to identify features of impending CeAD. The signs and symptoms of CeAD, which vary between vertebral and carotid artery dissection, commonly include headache and/or neck pain in 65-95% of patients and may also include cranial neuropathies, Horner's syndrome, pulsatile tinnitus, or other neurological manifestations [32]. CeAD can be challenging to detect due to its potentially benign-appearing symptoms, with a mean diagnostic delay of nine days (SD=12) [33]. Through an improved understanding of baseline risk, it remains possible that the time-to-diagnosis of this condition could be reduced, potentially improving patient outcomes.

Future research is needed to clarify the timing and mediating factors underlying the migraine-CeAD association. It remains unclear whether there is a close temporal association between migraine and CeAD, making it an ideal topic for a case-crossover (e.g., self-controlled) study. The potential association between migraine frequency, severity, and cumulative disease duration and CeAD should also be examined, potentially using a case-control design. This type of study may further help determine whether vascular anatomical variations, such as a hypoplastic vertebral artery [28], mediate the migraine-CeAD association. The potential influence of migraine management on CeAD risk also warrants investigation. A registry study with 1,468 CeAD patients found that migraineurs with CeAD were less likely to take beta-blockers compared to controls, suggesting a potential protective effect of certain medications used for migraine prevention [2]. While other medications were not associated with CeAD in that study within migraine subgroups (e.g., calcium channel blockers, tricyclic antidepressants, and antiepileptic drugs), several migraine-specific medications remain to be explored especially as pharmacologic management for migraines evolves.

Strengths and limitations

Strengths of this study include a large sample size, a registered protocol, and a comprehensive propensity matching strategy. However, several limitations may be noted. As a retrospective observational cohort study, our study design precludes any high certainty regarding causality between MwA or MwoA and migraine and only reports on associations between these conditions. The prevalence of migraine and CeAD may vary globally; thus, our findings may not generalize to regions outside of the United States. Unmeasured confounding may be present, for example, in relation to socioeconomic factors, health literacy, or access to imaging. Considering our eligibility criteria required non-migraine controls to have an initial examination, we cannot rule out the possibility of selection bias. However, our other eligibility criteria, including extensive exclusions of pre-existing cervical artery disorders, and matching strategy for several comorbidities and other factors aimed to attenuate this possibility.

TriNetX does not allow subgroup analyses within a given query. Additionally, as of July 9, 2025, the TriNetX network, which enables natural language processing, is no longer available. Therefore, further investigations into age subgroups are not possible with the present data. However, future studies using regression modeling could explore the potential influence of age on CeAD risk among migraineurs.

It would have been informative to describe the specific types of auras associated with patients in the MwA cohort, such as visual, sensory, or aphasic auras, or other clinical features such as migraine frequency, severity, or duration. However, TriNetX provides few granular details on these metrics, as migraine data are based predominantly on ICD-10 taxonomy codes. Accordingly, it remains possible that CeAD risk varies even within the MwA cohort depending on the migraine aura subtype and other clinical features. Follow-up studies could attempt to ascertain these valuable data points and use subgroup analysis or regression modeling to provide additional insights into the MwA-CeAD association.

While we are unaware of studies reporting the accuracy and positive predictive value of ICD-10 codes used specifically for MwA and MwoA, the positive predictive value of clinician-entered migraine diagnoses is high at 98% [34]. However, there is a possibility of misclassification of some included patients who initially were suspected of MwA or MwoA and instead had another primary or secondary headache disorder.

The positive predictive value of diagnosis codes for CeAD is 90% [35], and it is uncertain whether our natural language processing software would enhance the overall ascertainment of CeAD. Considering migraine is often chronic, further studies may be needed to examine longer follow-up windows. Our focus on new migraine diagnoses means that our findings may not apply to long-term migraine sufferers. Additionally, the impact of migraine therapies may influence CeAD likelihood, and further studies examining cohorts of migraineurs with varied medication regimens could be valuable.

## Conclusions

The present study supports our hypotheses that both MwA and MwoA are significantly associated with an increased risk of CeAD compared to non-migraine controls. These findings emphasize the need for heightened clinical awareness among clinicians who encounter patients experiencing migraines. Further research is necessary to explore the underlying pathophysiological mechanisms, the influence of migraine treatments, and the temporal relationship between migraines and CeAD.

## Figures and Tables

**Figure 3:**
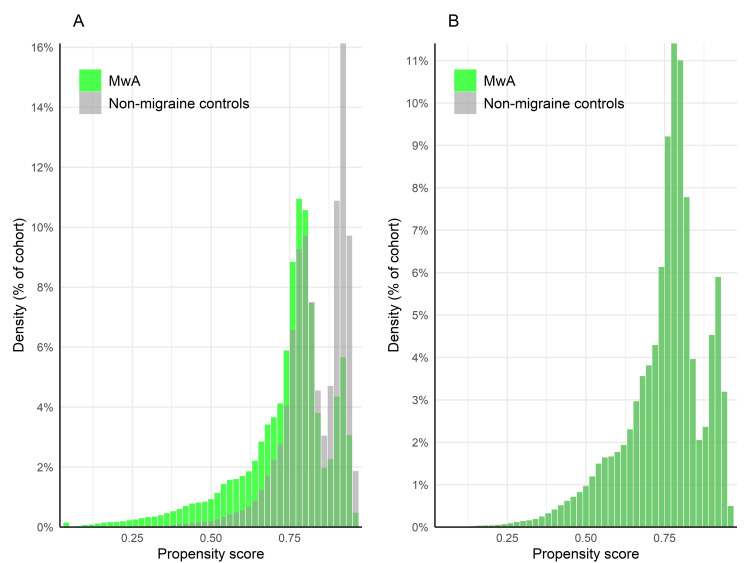
Propensity score density graph for MwA Density scores before (A) and after (B) matching. The green bars indicate the MwA cohort, while the gray bars represent the non-migraine control cohort. After matching, densities overlap closely, shown by darker green, suggesting sufficient balance of covariates. MwA: migraine with aura

**Figure 4:**
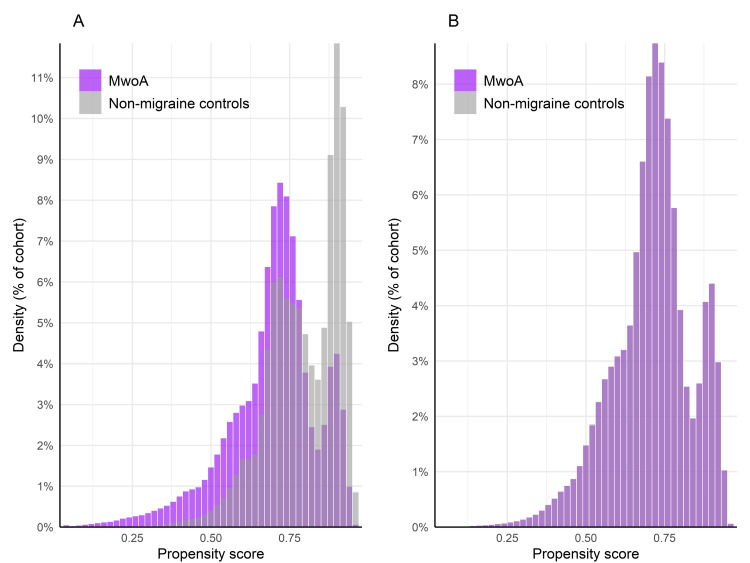
Propensity score density graph for MwoA Density scores before (A) and after (B) matching. The purple bars indicate the MwoA cohort, while the gray bars represent the non-migraine control cohort. After matching, densities overlap closely, shown by darker purple, suggesting sufficient balance of covariates. MwoA: migraine without aura

## Appendices

Appendix 1

**Table 3:** Exclusion criteria for cohorts -∞: any preceding duration of time available in the patient's data; *: TriNetX curated code; ATC: Anatomical Therapeutic Chemical Classification; CPT: Current Procedural Terminology; ICD-10: International Classification of Diseases, 10th Edition; ICD-10-PCS: ICD-10 Procedure Coding System; LOINC®: Logical Observation Identifiers Names and Codes; VA: Veterans Affairs National Drug File

ICD-10 codes	Definition	Window (days)
Exclusions for all cohorts		
Diagnoses		
I77.71 (ICD-10)	Dissection of the carotid artery	-∞ to 0
I77.74 (ICD-10)	Dissection of the vertebral artery	-∞ to 0
I77.75 (ICD-10)	Dissection of other precerebral arteries	-∞ to 0
S15.0 (ICD-10)	Injury of the carotid artery of the neck	-∞ to 0
S15.0 (ICD-10)	Injury of the vertebral artery	-∞ to 0
Procedures		
1022277 (CPT)	Transcatheter placement of extracranial vertebral artery stent(s), including radiologic supervision and interpretation, open or percutaneous	-∞ to 0
1022228 (CPT)	Transcatheter placement of intravascular stent(s), cervical carotid artery, open or percutaneous, including angioplasty, when performed, and radiological supervision and interpretation	-∞ to 0
35301 (CPT)	Thrombarterectomy, including patch graft, if performed; carotid, vertebral, subclavian, by neck incision	-∞ to 0
03QJ, 03QH, 03QN, 03QM, 03QL, 03QK (ICD-10-PCS)	Carotid artery repair	-∞ to 0
03QQ, 03QP (ICD-10-PCS)	Vertebral artery repair	-∞ to 0
Exclusions for examination cohorts only		
G43 (ICD-10)	Migraine	-∞ to 730
CN105 (VA)	Antimigraine agents	-∞ to 730
N02C (ATC)	Antimigraine preparations	-∞ to 730
64615 (CPT)	Chemodenervation of muscle(s); muscle(s) innervated by facial, trigeminal, cervical spinal and accessory nerves, bilateral (e.g., for chronic migraine)	-∞ to 730

Appendix 2

**Table 4:** Variables controlled for in propensity score matching CeAD: cervical artery dissection; ICD-10: International Classification of Diseases, 10th Edition; VA: Veterans Affairs National Drug File; *: custom curated TriNetX code

Variable	Description
Demographics	Age (at index and current age), sex
9083*	Body mass index (kg/m²)
9085*	Diastolic blood pressure (mm Hg)
9086*	Systolic blood pressure (mm Hg)
J00-J06	Acute upper respiratory infections
Z55-Z65	Adverse socioeconomic and psychosocial circumstances
E88.01	Alpha-1-antitrypsin deficiency
I71	Aortic aneurysm and dissection
I77.3	Arterial fibromuscular dysplasia
E06.3	Autoimmune thyroiditis
E08-E13	Diabetes mellitus
I70-I79	Diseases of arteries, arterioles, and capillaries (includes aneurysms, arterial embolism, arteritis, and arterial fibromuscular dysplasia)
Q79.6	Ehlers-Danlos syndromes
R79.82	Elevated C-reactive protein
R70	Elevated erythrocyte sedimentation rate and abnormality of plasma viscosity
D72.82	Elevated white blood cell count
Z82.4	Family history of ischemic heart disease and other diseases of the circulatory system
E72.11	Homocystinuria
E78.5	Hyperlipidemia, unspecified
I10-I1A	Hypertensive diseases
F10-F19	Mental and behavioral disorders due to psychoactive substance use
Q78.0	Osteogenesis imperfecta
Q87	Other specified congenital malformation syndromes affecting multiple systems (includes Marfan syndrome, Loeys-Dietz syndrome, Alport syndrome, arterial tortuosity syndrome)
O00-O99	Pregnancy, childbirth, and the puerperium
Z55-Z65	Persons with potential health hazards related to socioeconomic and psychosocial circumstances
Z72.0	Tobacco use
AM400 (VA)	Quinolones (includes fluoroquinolones)

Appendix 3

Appendix 4
